# A Novel Procedure for Preparing Mango Jellies with Higher Antioxidant Capacity and Reduced Sugar Content

**DOI:** 10.3390/ijms262110637

**Published:** 2025-10-31

**Authors:** Mladen Simonović, Milena Rašeta, Stefan Lekic, Darko Micic, Danica Savic, Djordje Nale, Ivan Vukovic, Maja Karaman, Annik Fischer, Nabil Adrar, Tuba Esatbeyoglu

**Affiliations:** 1 Institute of General and Physical Chemistry, IOFH, 11158 Belgrade, Serbia; micic83@gmail.com; 2 Department of Chemistry, Biochemistry and Environmental Protection, Faculty of Sciences, University of Novi Sad, 21000 Novi Sad, Serbia; milena.raseta@dh.uns.ac.rs; 3 Institute of Chemistry, Technology and Metallurgy, IHTM, 11000 Belgrade, Serbia; stefan.lekic@ihtm.bg.ac.rs (S.L.); danica.savic@ihtm.bg.ac.rs (D.S.); 4 Clinic of Urology, University Clinical Centre of Serbia, 11000 Belgrade, Serbia; djordje.nale@gmail.com (D.N.); ivanvukovic.urolog@gmail.com (I.V.); 5 ProFungi Laboratory, Department of Biology and Ecology, Faculty of Sciences, University of Novi Sad, 21000 Novi Sad, Serbia; maja.karaman@gmail.com; 6 Department of Molecular Food Chemistry and Food Development, Institute of Food and One Health, Leibniz University Hannover, 30167 Hannover, Germany; fischer@foh.uni-hannover.de (A.F.); adrar@foh.uni-hannover.de (N.A.)

**Keywords:** *Mangifera indica* L., DC polarography, antioxidant activity, bioactive compounds, hypoglycemic activity

## Abstract

This study investigated the impact of two different temperature regimes (high and low) on the chemical composition, antioxidant activity, and antidiabetic properties of mango jellies. Total phenolic content (TPC) and antioxidant capacity were assessed using the conventional 2,2-diphenyl-1-picrylhydrazyl (DPPH) assay and the recently developed direct current (DC) polarographic method. Jellies prepared under low-temperature conditions contained higher TPC levels (82.0 ± 2.0 mg gallic acid equivalents (GAE)/kg jelly) and exhibited stronger antioxidant activity (65.0 ± 2.2 ascorbic acid equivalents (AAE)/100 g jelly by DPPH; 12.40 × 10^−6^ mol reduced Hg(II)/g jelly by DC polarography). Antidiabetic evaluation revealed that the low-temperature jelly significantly inhibited both α-amylase and α-glucosidase activity. Thermal analysis further confirmed distinct structural behavior between low- and high-temperature products. This novel processing approach—combining mild heating (55 °C), vacuum treatment, and reduced sugar content (40%) without pectin addition—proved effective in preserving bioactive compounds and functionality. Notably, this is the first report applying DC polarography to assess antioxidant capacity in fruit jellies, highlighting its potential as a robust tool in functional food research.

## 1. Introduction

Mango (*Mangifera indica* L.), often referred as the “food of the Gods” or the “king of fruits”, is one of the most widely consumed tropical fruits worldwide [1,2,3]. Mango’s bioactive compounds are strongly linked to beneficial effects on human health due to their nutritional importance and value [2,3,4]. Major bioactive compounds identified in mango include phenolic acids, flavonoids, carotenoids and vitamins (notably niacin, riboflavin, ascorbic acid, and thiamine) [3,5,6,7]. Mango exhibits significant antioxidant activity, largely attributed to its rich content of phenolic compounds such as mangiferin, gallic acid, and quercetin [8]. Studies on mango leaf extracts have shown moderate to strong radical scavenging and metal-reducing capacities across multiple assays (DPPH, 2,2′-azino-bis(3-ethylbenzothiazoline-6-sulfonic acid) (ABTS), Ferric Reducing Antioxidant Power (FRAP), Cupric Ion Reducing Antioxidant Capacity (CUPRAC)), highlighting their potential in neutralizing oxidative stress [9]. Mangiferin, a major xanthone in mango, contributes substantially to this effect through scavenging reactive oxygen species and chelating metal ions, supporting its therapeutic role in managing oxidative damage in neurodegenerative and metabolic diseases [10]. Furthermore, mango bark and leaf extracts demonstrate considerable total phenolic and flavonoid content, correlating with significant antioxidant capacities measured by DPPH and FRAP assays, affirming their value as natural antioxidant sources [11]. The overall antioxidant efficacy arises not only from individual phenolics but also synergistic interactions among bioactive compounds [12,13], supporting the use of mango in food, nutraceutical, and pharmaceutical applications. Use of isolated compounds has often resulted in weaker effects compared to whole-fruit consumption, suggesting that mango polyphenols act synergistically to maximize antioxidant activity [14]. Among these, mangiferin is particularly noteworthy, as its antioxidant activity also modulates inflammatory processes, including those associated with infection and diabetic conditions [15,16,17]. Beyond antioxidative effects, mango consumption has been linked to relief from constipation [18], prevention of cancer [19,20], and significant roles in managing diabetes mellitus (DM) [21,22,23], cardiovascular disease, and inflammatory processes [24].

Mango is consumed fresh, in fruit salads, or in processed products such as juices and jellies [25,26,27]. However, industrial processing often involves heat treatments that can degrade heat-labile bioactive compounds (phenolics, flavonoids, carotenoids, vitamins), with losses depending on temperature and processing time [28]. Despite the wide use of mango in processed foods, mango jellies in particular have not been systematically investigated for their functional food potential, representing an important knowledge gap.

In this study, we aimed to develop low-sugar mango jellies with high fruit content and improved retention of bioactive compounds. Our approach focused on producing a gel without pectin, in contrast to recent studies that required pectin as a gelling agent [29].

In addition to these health-oriented motivations, current global market trends strongly support the development of reduced-sugar and functional jelly products. The global jelly market is projected to grow from USD 4.15 billion in 2024 to USD 6.58 billion by 2033, with a compound annual growth rate (CAGR) of 5.24% [30]. Within this sector, the low-calorie jelly market—valued at approximately USD 3.61 billion in 2025—is expected to reach USD 6.46 billion by 2035, driven largely by the incorporation of natural sweeteners such as stevia, monk fruit, and erythritol [31]. Similarly, the sugar-free jelly segment, valued at around USD 1.2 billion in 2021, is projected to nearly double to USD 2.3 billion by 2028 [32]. Consumer preferences are also shifting toward formulations enriched with natural ingredients, such as apple juice concentrate and honey, as alternatives to refined sugars [33]. These market dynamics highlight the growing demand for healthier, low-sugar jelly formulations and underscore the practical relevance of our study.

Attempts to prepare mango gels with added gelatin have been unsuccessful because mango (like many tropical fruits) contains a wide range of proteases that interfere with gel formation [34].

Here, we introduce an innovative processing method combining higher fruit content, lower temperatures, and vacuum conditions to achieve a stable gel-like product at laboratory scale. This work was designed not only to examine the antioxidant potential of mango jellies but also to evaluate their antidiabetic activity through α-amylase and α-glucosidase inhibition, and to assess their thermal properties. To the best of our knowledge, this is the first investigation of mango jelly as a potential functional food, linking its processing conditions with antioxidant, antidiabetic, and structural characteristics.

## 2. Results and Discussion

### 2.1. Biological Properties of Jellies

The jellies prepared under low-temperature regime (M2 and M3) contained higher total phenolic content (TPC) and exhibited greater antioxidant potential compared to the jelly produced at high-temperature regime (M1) (Table 1).

The higher TPC could explain why the antioxidant values (Table 2) and α-amylase/α-glucosidase inhibition activities (Table 3) were enhanced in the low-temperature jellies compared to the high-temperature jelly (M1), indicating a potential for slower glucose absorption through inhibition of these enzymes.

#### 2.1.1. Phenolic Profile and Antioxidant Activity

The low-temperature jellies (M2: 82.0 ± 2.0 mg GAE/kg; M3: 80.4 ± 1.9 mg GAE/kg) had significantly higher TPC than the high-temperature jelly (M1: 70.0 ± 3.7 mg GAE/kg), as indicated by different superscript letters (a vs. b). Similarly, DPPH values were significantly higher for M2 (65.0 ± 2.2 mg AAE/100 g) and M3 (63.3 ± 3.1 mg AAE/100 g) compared to M1 (53.0 ± 3.4 mg AAE/100 g) as shown in Table 1.

During jelly preparation at atmospheric pressure and high temperature, the TPC in the final product was reduced by up to 15% compared to products made under the low-temperature regime. This clearly highlights the advantage of mild, non-invasive processing conditions for mango jelly production. Furthermore, the TPC values obtained in this study were consistent with previously published data, i.e., 33–120 mg GAE/kg [35] and 18–140 mg GAE/kg [36].

A direct link between TPC and antioxidant activity has been extensively reported in the literature [37,38,39]. Phenolic profiling of mango (Table S1) has revealed a broad diversity of compounds, with individual studies identifying between 4 and 16 phenolics depending on the tissue analyzed (pulp, peel, seed kernels, or leaves) and the analytical method used. Among these, chlorogenic acid, gallic acid, protocatechuic acid, vanillic acid, mangiferin, and quercetin derivatives are recurrently reported as major constituents across different mango parts. Chlorogenic acid, gallic acid, vanillic acid, and protocatechuic acid are considered major phenolics, with their content shown to increase during fruit ripening [40]. Another report indicates that gallic acid content ranges from 2.3 to 25.62 mg/100 g in mango peel and seed kernel, while mangiferin is a dominant phenolic, especially in peel extracts, with detailed quantification provided. Although direct studies on mango jelly phenolics are lacking, the work of Lebaka et al. [41] quantified mangiferin (≈22.15 mg/100 g fresh weight in peel) and gallic acid across mango cultivars, data which are relevant for mango-based products including jellies. These phenolics vary with cultivar and ripening stage and contribute to the nutritional and antioxidant properties of mango. Notably, research on mango peel polyphenols demonstrated that addition of mango peel powder to jelly formulations increased carotenoid content by 1.3–1.6-fold and phenolic compounds by 3.2–4.8-fold, although some darkening of the jelly was observed. Also, phenolic compounds are better preserved when processed at lower temperatures, and the values obtained here differ only slightly from those reported in recent studies [42]. Investigations on fresh versus heated fruits have demonstrated that quantifiable phenolics decrease significantly even at 60 °C [43].

In mango, thermal treatment was shown to strongly reduce citric acid, whereas gallic acid and mangiferin were largely unaffected [44]. Other authors [45] reported a marked decline of gallic acid after hot-water treatment, but more recent studies [46] indicated stable levels after heating. Citric acid contributes not only to antioxidant activity but also to flavor and color retention in mango pulp [47]. Preservation of gallic acid is important because of its antiradical activity as well as its antibiotic, prebiotic, and antiproliferative effects [48,49,50]. Mangiferin, a C-glucosyl xanthone, is also a potent antioxidant that neutralizes reactive oxygen species (ROS), thereby protecting pancreatic β-cells, preserving insulin secretion, and exerting additional anti-inflammatory and prebiotic effects [46,51]. Regarding the mechanistic explanation of these results, operating under vacuum conditions significantly reduces oxygen exposure [52], thereby limiting oxidative degradation and enzymatic browning reactions. Second, mild heating at 55 °C inhibits the activity of oxidative enzymes such as polyphenol oxidase and peroxidase, which are highly sensitive to both temperature and oxygen availability. In contrast, high-temperature processing promotes the denaturation of phenolics and facilitates non-enzymatic oxidation, resulting in lower total phenolic content [53]. The reduced oxygen partial pressure and controlled heat transfer during vacuum processing thus contribute to the stabilization and retention of heat-labile phenolic antioxidants, consistent with the higher TPC and antioxidant activity detected in the low-temperature jellies.

Jelly preparation in this study avoided separation of juice and pulp. Instead of boiling the fruit at >80 °C, a low-temperature regime (55 °C) was applied in the subsequent step. Notably, mango jellies were prepared without pectin addition, enabled by the high fruit content (60%), compared to the standard minimum of 35%. Literature reports indicate that for low-fruit formulations, pectin (0.5–1.5%) must be added and pH adjusted with citric acid to 3.0–3.5 to achieve gel formation [54]. To the best of our knowledge, this is the first investigation describing the production of mango jellies completely without pectin.

In parallel, this work is also the first to apply the recently developed DC-polarography method for determining the antioxidant potential of fruit jellies. Antioxidant activity measured by DC-polarography was significantly higher in M2 (12.40 × 10^−3^ ± 3.0 × 10^−8^ mol Hg(II)/kg) and M3 (11.84 × 10^−3^ ± 3.0 × 10^−8^) than in M1 (3.74 × 10^−3^ ± 2.6 × 10^−8^), with high reproducibility (R^2^ > 0.97) (Table 2).

The experimental data demonstrated an excellent correlation between the results of the DPPH (Table 1) and DC-polarography assays (Table 2), reinforcing the robustness of both methods. Unlike spectrophotometric assays, DC-polarography directly measures the intrinsic reducing power of redox-active compounds, thereby detecting antioxidants that may not react efficiently with stable free radicals such as DPPH. Moreover, this electrochemical technique offers very high sensitivity and low detection limits, which is particularly relevant when processing conditions leave only trace amounts of heat-labile compounds. A further advantage is the reduced influence of matrix effects such as pigments or turbidity, which often interfere with spectrophotometric methods, making DC-polarography especially suited for complex fruit-based products like jellies. In addition, the ability to monitor multiple redox-active species simultaneously provides complementary information on the antioxidant profile, rather than only a single aggregated value. According to the results presented in Table 2, the antioxidant activity did not differ significantly between the M2 and M3 samples, which is consistent with the DPPH assay results (Table 1), where both M2 and M3 emerged as the most promising samples. Importantly, the strong correlation observed between DPPH and DC-polarography confirms that the electrochemical approach is both reliable and robust. By integrating this method, our study broadens the methodological framework for antioxidant assessment and demonstrates that DC-polarography, while underutilized, provides unique advantages that justify its application in functional food research.

#### 2.1.2. Antidiabetic Activity

The inhibitory effects of mango jellies against carbohydrate-hydrolyzing enzymes are summarized in Table 3. For α-amylase, acarbose exhibited the strongest inhibition (95.52 ± 4.62%), while among the jellies, M2 demonstrated moderate activity (37.50 ± 2.11%), significantly higher than M3 (1.10 ± 0.15%) and M1 (inactive). These results suggest that only the low-temperature jelly without pectin (M2) exhibited relevant α-amylase inhibition, whereas the addition of pectin (M3) drastically reduced this effect.

Acarbose exhibited a 95.52% inhibition of α-amylase activity, outperforming all mango extracts (Table 3). However, the M2-jelly extract demonstrated moderate but significant activity (37.50%), suggesting potential utility in managing postprandial hyperglycemia. For α-amylase, the inhibition was very low for M3-jelly (1.10 ± 0.15%), while the extract of M1-jelly showed no activity. The explanation for why M3-jelly (with added citrus pectin) showed lower α-amylase inhibition compared to the same jelly without pectin (M2) may lie in the dual role of pectin. Pectin can have either inhibitory or enhancing effects on α-amylase activity depending on its structure and interactions. This may be due to pectin’s ability to alter the physicochemical environment of amylase, in some cases making it more active [55]. A more recent study also found that pectin increased α-amylase activity in processed food retrieved from the digestive system of grower pigs [56].

With respect to mango itself, one recent study demonstrated that the seed kernel extract (2.67 to 9.83 mg/mL) exhibited the highest α-amylase inhibitory activity compared to peel extract. Therefore, the seed kernel of *Mangifera odorata* has been recognized as an excellent candidate for use as a natural hypoglycemic agent for diabetes management, offering fewer side effects than acarbose or other synthetic drugs [57].

In contrast to α-amylase, the inhibition of α-glucosidase followed a different trend. The M2-jelly extract showed moderate activity (32.63%), M1 again showed no activity, and M3-jelly (with added citrus pectin) exhibited the strongest effect, even surpassing Acarbose (92.88% vs. 86.26%). Recent studies confirm that pectin, especially from citrus and similar fruits, can inhibit α-glucosidase by slowing the breakdown of carbohydrates, which subsequently reduces the rate of sugar absorption into the bloodstream [58].

Taken together, the results suggest that while the addition of pectin diminished α-amylase inhibition, it enhanced α-glucosidase inhibition. This dual influence of pectin can be explained by its molecular interactions with both the enzyme and the starch substrate [59]. On one hand, pectin increases the viscosity of the surrounding medium, restricting substrate diffusion and limiting α-amylase access to starch granules [60]. In addition, pectin molecules may bind to α-amylase via hydrogen bonds and electrostatic interactions, inducing conformational changes that reduce catalytic efficiency [60]. Conversely, certain low-methoxyl pectins form soluble complexes with starch, enhancing substrate accessibility and facilitating partial hydrolysis [61].

According to the available literature data, this is the first study to demonstrate the ability of mango jellies to inhibit α-amylase. In addition, by linking our findings with mango’s phenolic composition (Table S1), it becomes clear that compounds such as mangiferin, quercetin derivatives, gallic acid, and vitamin C are strongly implicated in α-glucosidase and α-amylase inhibition [62,63]. Selim et al. [64] reported that mango leaf extracts exhibiting the highest inhibition of α-amylase activity did not necessarily produce significant inhibition of α-glucosidase activity, indicating selective enzyme inhibitory effects of the extracts.

Notably, cinnamic acid, also identified in mango samples (Table S1), has been recognized for its role in enhancing glucose tolerance and stimulating insulin secretion in response to glucose [13]. The combined action of these bioactive compounds, together with the structural effects of dietary fibers like pectin, underpins the inhibitory potential of mango jellies on carbohydrate-hydrolyzing enzymes. This provides a biochemical rationale for the observed in vitro antidiabetic activity and supports the concept of mango jelly as a promising functional food for managing postprandial hyperglycemia.

#### 2.1.3. Principal Component and Pearson Correlation Analyses

**Figure 1:**
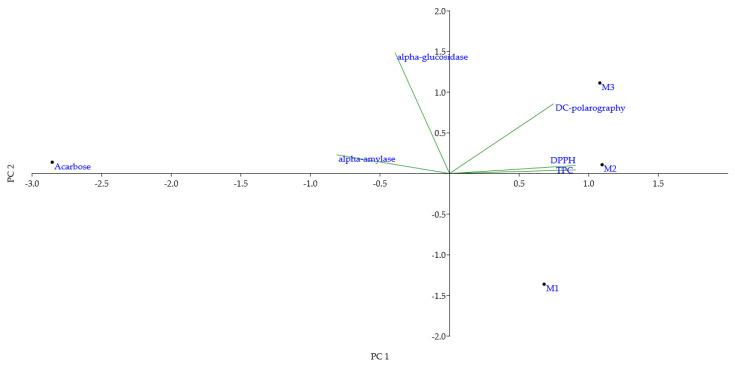
Principal component analysis (PCA) of all examined parameters in three tested mango jellies (M1–M3) and the standard compound, acarbose. Abbreviations: DPPH—radical scavenger capacity against 2,2-diphenyl-1-picrylhydrazyl radical, DPPH^⋅^; TPC—total phenolic content.

To integrate the results, Principal Component Analysis (PCA) was performed (Figure 1).

The first two components explained 94.03% of the total variance (PC1 = 73.20%; PC2 = 20.83%), demonstrating that most of the data variability was captured. TPC and DPPH clustered on the positive side of PC1, whereas DC-polarography was located on the opposite side, indicating that electrochemical measurements provided distinct but complementary information on antioxidant behavior. The enzyme inhibition variables (α-amylase, α-glucosidase) were positioned separately from antioxidant parameters, consistent with their different underlying mechanisms. Sample distribution reflected these relationships: M3 was closely aligned with α-glucosidase inhibition, confirming its strong inhibitory effect observed in Table 3; M2 displayed a balanced profile across antioxidant and enzyme inhibitory activities; and M1 showed minimal association with either antioxidant or antidiabetic properties.

Pearson’s correlation analysis (Table S2) further supported these findings. A strong positive correlation was observed between TPC and DPPH, whereas no significant correlation was detected between phenolic content and enzyme inhibition. These outcomes confirm that antioxidant preservation and antidiabetic potential are related but independent attributes of mango jellies, highlighting the importance of evaluating both types of functionality in product development.

### 2.2. Sensory Evaluation of Jellies

The results of sensory analysis are shown in Table 4. For taste, no significant differences were observed among the jelly samples, indicating that pectin addition did not influence this parameter. This aligns with prior findings that pectin inclusion typically does not alter flavor in fruit-based gels [60]. Spreadability was significantly reduced in the pectin-containing sample M3 compared to M1 and M2. This is consistent with the known effect of pectin increasing gel viscosity and firmness, which can limit spreadability in jellies and similar products, as supported by studies on pectin’s impact on texture and its molecular interactions affecting enzyme activity and gel structure [65].

Gelation was significantly enhanced in M3, the jelly with 1% pectin, confirming pectin’s role as an effective gelling agent that strengthens the gel matrix. Such effects have been widely documented, demonstrating pectin’s ability to form stable networks in fruit gels [66].

Overall, the low-temperature vacuum-processed jelly without pectin (M2) received the highest overall sensory scores, although differences with the other samples were not statistically significant. This indicates that gentle thermal processing can preserve sensory quality effectively, in agreement with studies showing that low-temperature treatments help retain both quality and sensory attributes in fruit products [67,68]. To balance gel strength and spreadability, optimizing pectin concentration or blending pectin with other hydrocolloids may be advantageous, as demonstrated in several studies where mixed hydrocolloid systems improved textural properties while maintaining consumer appeal [69]. Taken together, these findings highlight that while pectin enhances gelation, low-temperature vacuum processing without pectin (M2) offers the best balance of desirable sensory properties, suggesting a promising pathway for producing clean-label mango jellies with both good texture and consumer acceptance.

### 2.3. Thermal Analysis of Jellies

**Figure 2:**
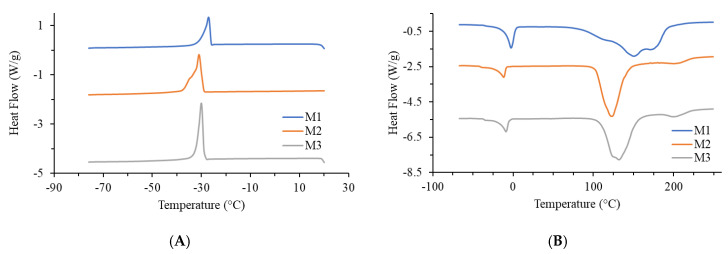
DSC thermograms of (**A**) cooling and (**B**) heating mango jellies M1–M3. Cooling/heating rate was 5 °C/min, and nitrogen flow was 50 mL/min. Abbreviations: M1—Jellies prepared under high-temperature regime (boiling before and after sugar/calcium citrate addition); M2—Jellies prepared under low-temperature vacuum regime (55 °C, with calcium citrate, no pectin); M3—Jellies prepared as M2, but with 1% pectin added. Each DSC analysis was performed once per sample, as the thermal measurements are destructive and cannot be repeated on the same specimen.

The cooling (A) and heating (B) DSC curves of the analyzed mango jelly samples are shown in Figure 2, and the parameters determined from the curves are listed in Table 5. All three samples were analyzed: M1 (mango jelly prepared by classic procedure with heating), M2 (mango jelly prepared at low temperature, without pectin), and M3 (mango jelly prepared at low temperature, but exceptionally with added pectin). Among them, the M3 sample was specially made only to see whether the high temperature or pectin would cause any differences in thermal behavior of the final product.

Thermal analysis was included to evaluate how different processing regimes affect the stability and molecular organization of the jelly matrix, since thermal events such as glass transition, water binding, and decomposition are directly linked to product quality, shelf life, and texture. By comparing M1, M2, and M3, it was possible to assess whether low-temperature processing better preserves structural integrity compared to conventional heating. During the cooling phase, all three samples exhibited an exothermic peak with a temperature of around −22 °C for the M1 sample, and around −30 °C for the M2 and M3 samples. This peak occurred as a result of water crystallization present in the samples. Upon heating, two endothermic peaks were detected in all three samples. The first smaller peak, with a temperature in the range of −11.7 °C to −2.6 °C, resulted from the melting of water. The second peak, which began between 102 °C and 112 °C, was significantly broader and more complex; the M1 sample exhibited three temperature values, while the M2 and M3 samples had two temperature values each. This peak was due to the thermal effect from water evaporation and the onset of degradation of organic macromolecules present in the samples. Because of the overlap of energetic effects in the same temperature range, complex peaks appeared on the DSC curve in such intricate systems as jelly. Based on the shape of this peak, it can be concluded that the M2 and M3 samples have very similar thermal characteristics, while the M1 sample differs significantly from them.

**Figure 3:**
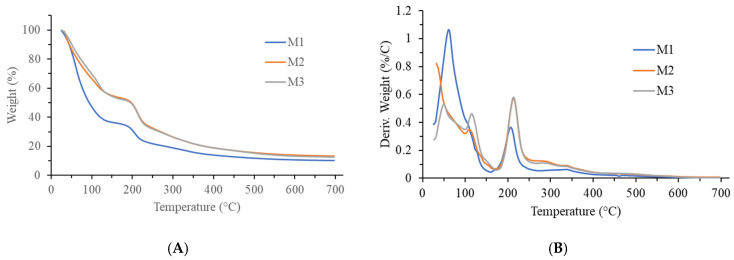
(**A**) Thermogravimetric (TG) and (**B**) differential TG (dTG) curves of mango jellies M1-M3. Heating rate was 5 °C/min, and nitrogen flow was 60 mL/min. Abbreviations: M1—Jellies prepared under high-temperature regime (boiling before and after sugar/calcium citrate addition); M2—Jellies prepared under low-temperature vacuum regime (55 °C, with calcium citrate, no pectin); M3—Jellies prepared as M2, but with 1% pectin added. Each TG analysis was performed once per sample, as the thermal measurements are destructive and cannot be repeated on the same specimen.

Thermogravimetric (TG) and differential TG (dTG) curves of the analyzed samples are presented in Figure 3, with the results shown in Table 5. All three samples exhibited weight loss in two steps (two peaks on the dTG curves). The first step, occurring from room temperature to approximately 170 °C, was due to the loss of water and other volatile components present in the samples. It accounted for 64% for the M1 sample, and around 46–47% for the M2 and M3 samples. It was also observed that the peak in the M2 and M3 samples was more complex, showing two temperature values, in contrast to the M1 sample, where only one temperature was observed. This may be because the M2 and M3 samples contained two types of water: weakly bound water, which evaporated at lower temperatures, and strongly bound water, which required higher temperatures to evaporate. It is well known that pectin has strong water-binding properties, but there is also the fact that protein hydration is crucial for their native, three-dimensional structure. In the samples M2 and M3 these molecules are well preserved, while upon bringing in the sample M1 to boiling point the proteins were unfolded and the pectin is partially broken down making the water easier to evaporate [70,71].

The second step of weight loss occurred in the range of approximately 160–170 °C to around 400 °C for all three samples and can be attributed to the thermal decomposition of biological material/macromolecules present in the samples. On the dTG curves, it was characterized by a peak with a broad shoulder towards higher temperatures, indicating that the process was complex. This is understandable given the complex composition of the analyzed jellies. The weight loss in this stage was around 22% for the M1 sample, while it was approximately 33–34% for the M2 and M3 samples. The residue at 700 °C was around 10% for the M1 sample and approximately 13% for the M2 and M3 samples. Based on the shape of the TG curves, it can also be concluded that the M2 and M3 samples were quite similar in thermal properties, in contrast to the M1 sample, which differed significantly from them.

Taken together, these findings suggest that low-temperature processing (M2 and M3) preserves molecular integrity and water-binding capacity more effectively than high-temperature boiling (M1). Thermal analysis, although secondary to our bioactivity assays, provided valuable insight into how processing influences jelly structure and stability, reinforcing our conclusion that mild processing better maintains functional quality. This is consistent with previous report [72] showing that low-temperature processing of mango minimizes enzymatic degradation and metabolic loss, while preserving membrane and cell wall integrity. In contrast, high heat treatments typically reduce firmness, induce structural alterations, and impair water-binding through protein denaturation and membrane permeabilization.

### 2.4. Limitations of the Study

This study has several limitations that should be acknowledged. First, the experiments were conducted on a limited number of mango jelly formulations prepared under controlled laboratory-scale conditions, which may not fully reflect the variability encountered in industrial production. Second, the analysis was restricted to selected antioxidant assays and α-amylase/α-glucosidase inhibition, while other bioactivities potentially relevant to functional foods were not assessed. Third, molecular-level mechanisms underlying the observed effects were not explored in depth; therefore, further studies, including metabolomic or proteomic approaches, are warranted to clarify the precise pathways involved. Finally, while basic sensory attributes were assessed, broader consumer acceptability testing was not performed, which remains a critical step for future product development and application.

## 3. Materials and Methods

### 3.1. Chemicals

All chemicals used were of analytical grade quality, unless otherwise specified. H_3_BO_3_, KCl, HgCl_2_ and NaOH were purchased from Merck (Darmstadt, Germany). Gaseous nitrogen of high purity (>99.995%), which was used to remove dissolved oxygen from cell solution, was purchased from Messer, Serbia. 96% ethanol was commercially obtained from Reahem, Serbia. Methanol, Folin–Ciocalteu reagent, gallic acid, DPPH radical and vitamin C were purchased from Sigma Aldrich (St. Louis, MI, USA). Citrus LM Pectin was purchased from Carbosynth, Compton, Berkshire, UK. α-amylase, α-glucosidase, starch, acarbose and p-nitrophenyl α-D-glucoside were purchased from Sigma-Aldrich (Steinheim, Germany). Lugol’s iodine solution was purchased from Carl Roth GmbH + Co. KG (Karlsruhe, Germany). Na_2_CO_3_, HCl, Na_2_HPO_4_ and NaH_2_PO_4_ were purchased from Betahem, Belgrade, Serbia.

### 3.2. Fruit Samples and Preparation of Mango Jellies

Fruit samples of mango (*Mangifera indica* L., cv. Keitt), originating from Brazil, were purchased at a local supermarket in Serbia. The fruits were cut in half, the seeds removed, and the pulp was separated from the juice by passing it through a cloth [73]. On average, one fruit weighed approximately 400 g, yielding about 250 g of edible pulp after removing the peel and seed. One mango fruit was used to prepare one jelly sample.

**Figure 4:**
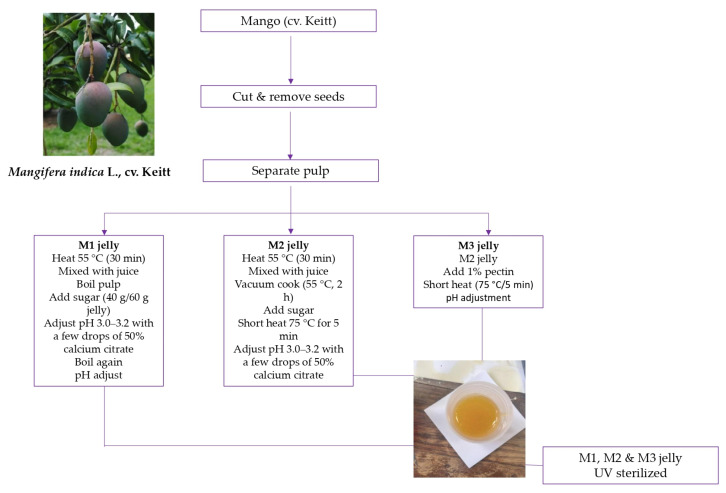
Schematic illustration of the preparation of M1, M2, and M3 mango jellies.

For jelly preparation (Figure 4), the pulp was first heated at 55 °C for 30 min to achieve a more fluid state.

The fluid pulp was then mixed with juice and cooked under vacuum (≈−900 mbar) at a low temperature (55 °C) for 2 h until the dry matter content reached 33% [74]. Sugar was subsequently added until the dry matter increased to 60% (40 g sugar per 60 g jelly). After short heating (5 min at 75 °C), the pH was adjusted to 3.0–3.2 by adding a few drops of 50% calcium citrate solution [74], which was necessary to obtain the product in a gelled state. The jellies were then poured into glass jars sterilized overnight with UV light and labeled as M2.

For comparison, jellies were also prepared under a high-temperature regime, in which the fruit contents were brought to the boiling point both before and after the addition of sugar and calcium citrate. These samples were designated as M1.

Additionally, in order to investigate whether the addition of pectin could influence the thermal properties or antidiabetic potential, a third type of jelly was produced in the same way as M2, but with added pectin at a final concentration of 1%. These jellies were labeled as M3.

### 3.3. Determination of Jelly Moisture and Total Soluble Contents

The moisture of the jellies was determined by drying 1.0 g of fresh samples at 105 °C for 2 h, cooling in a desiccator, and weighing. The percentage of water (moisture) was determined by subtracting the dry weight from the initial weight. Thus, %Total solids = (100% − %Moisture).

### 3.4. Preparation of Samples for Analysis

About 1 g of jelly sample was weighed and extracted with 5 mL of methanol in an ultrasonic bath (LEO Ultrasonic Co., Ltd., New Taipei City, Taiwan) for 15 min at 25 °C. The extracts were then centrifuged (Eppendorf^®^ Centrifuge 5427R, Hamburg, Germany), and the supernatants were collected and stored at 4 °C until further analyses.

### 3.5. Determination of Total Phenolic Content

Phenolic extracts of the jellies were prepared by methanol extraction following the procedure described in Section 3.4. Total Phenolic Content (TPC) was determined according to the Folin–Ciocalteu method by using a spectrophotometer (Evolution 600, Thermo Fisher, Waltham, MA, USA) [75]. Gallic acid was used as a calibration standard, while the results are expressed as milligrams of gallic acid equivalents (GAE) per kg of the jelly (mg GAE/kg jelly).

### 3.6. Determination of Antioxidant Activity by the DPPH Spectrophotometric Method

Samples for DPPH assay were prepared as described in Section 3.4. The method was based on the reaction with the stable radical DPPH [76]. A solution of DPPH radical in methanol (0.04 mg/mL) was freshly prepared, and vitamin C (ascorbic acid) was used as the reference standard. For each measurement, 200 μL of sample extract was mixed with 1800 μL of DPPH radical solution. The mixtures were incubated in the dark for 30 min, after which the absorbance was measured at 517 nm (Evolution 600, Thermo Fisher, Waltham, MA, USA). The results for antioxidant capacity (VCEAC) were expressed as mg of ascorbic acid equivalents (AAE) per 100 g of the jelly (mg AAE/100 g jelly).

### 3.7. Direct Current (DC)-Polarographic Method for the Determination of Antioxidant Activity

For DC-polarographic method, 20 mg of jelly sample was weighed and extracted with 1 mL of 80% ethanol by vigorous shaking for 1 h at room temperature. After that, the samples were centrifuged at 5000 rpm for 10 min (Eppendorf^®^ Centrifuge 5427R, Hamburg, Germany).

In the polarographic vessel, 10.0 mL of Clark and Lubs buffer pH 9.8 and 200 µL of 1.00 × 10^−2^ M HgCl_2_ solution were added. Clark & Lubs buffer was prepared from a solution containing 0.20 M of both H_3_BO_3_ and KCl, to which 0.20 M NaOH was added up to desired pH. The solution was stirred and deaerated for 5 min with nitrogen 5.0 and three polarograms in the absence of sample were recorded. Then several equal sample aliquots (100 µL) were added. After adding every aliquot, the solution was stirred and deaerated with nitrogen for 30 s and three polarograms were recorded. Polarograms were recorded between 0.15 and −0.25 V vs. the reference electrode.

As the measure of the antioxidant activity, the slope of the initial, linear part of the dependence of the polarographic wave of the HgCl_2_ reduction height on the volume of the sample added was used. Antioxidant activity was expressed as the mols of Hg^2+^ reduced per gram of sample.

The instrument used was Autolab PGSTAT101 connected with Autolab IME663 interface to Metrohm 663 VA Stand equipped with Multi-Mode Electrode pro in the SMDE mode as working, a glassy carbon electrode as auxiliary, and 3 M Ag/AgCl as reference electrode. The height of the Hg(II) chloride reduction wave was measured at 0 V vs. the reference electrode [77].

### 3.8. In Vitro Antidiabetic Potential

#### 3.8.1. α-Amylase Inhibitory Activity

The in vitro antidiabetic potential, reflected by a hypoglycemic effect, was evaluated by testing the ability of the extracts to inhibit the digestive enzyme α-amylase. The α-amylase inhibitory activity was measured according to Yang et al. [78]: 90 μL of α-amylase (porcine pancreas Type VI–B, glucose) was combined with 80 μL of a 0.05% starch solution prepared in 20 mM phosphate buffer (pH 6.9) and 10 μL of the extract (10 mg/mL) or acarbose standard (0.016–0.5 mg/mL). For the blank sample, α-amylase was replaced with a phosphate buffer (pH 6.9), while in control samples, the extract or standard were substituted with the same buffer. The mixtures were incubated at 37 °C for 10 min continuously shaking (IKA KS 4000i control, IKA-Werke GmbH & Co., KG, Staufen, Germany). The reaction was terminated by adding 100 μL of chilled 1 M HCl and 20 μL of Lugol’s iodine solution, and the absorbance was read at 620 nm. All assays were conducted in triplicate, and the results were presented as the percentage of enzyme inhibition.

#### 3.8.2. α-Glucosidase Inhibitory Activity

The α-glucosidase inhibition assay was performed following the method described by Palanisamy et al. [79], with slight modifications. In brief, a reaction mixture was prepared by combining 100 μL of 0.1 M phosphate buffer (pH 6.8), 10 μL of α-glucosidase enzyme (from *Saccharomyces cerevisiae*, Type I), 20 μL of the test extract (11.11 μg/mL) or the standard acarbose (at concentrations ranging from 0.03 to 1.11 mg/mL), and 20 μL of p-nitrophenyl α-D-glucoside. For the blank sample, the enzyme was replaced with a phosphate buffer (pH 6.8), and for the control, the extract or acarbose was replaced with 20 μL of the same buffer. The mixtures were incubated at 37 °C for 15 min with continuous shaking using an IKA KS 4000i control shaker (IKA-Werke GmbH & Co., KG, Staufen, Germany). Following incubation, 80 μL of 0.2 M (Na_2_CO_3_, Betahem, Belgrade, Serbia) was added to stop the reaction, and the absorbance was recorded at 400 nm. All assays were conducted in triplicate, and the results were reported as the percentage of enzyme inhibition.

### 3.9. Sensory Evaluation of the Mango Jellies

Sensory evaluation of the jelly samples was conducted using a randomly selected panel of 20 volunteers (80% male, aged 19–64 years), all patients of the Clinic of Urology, University Clinical Centre of Serbia (Belgrade, Serbia). The panelists were not trained experts; they received verbal instructions describing the procedure and the sensory descriptors to be evaluated. Participants had previous experience with similar fruit products only if they were commercially purchased.

The sensory attributes assessed included taste, spreadability, and gelation. The volunteers were kindly asked to taste each jelly sample and evaluate it using a 5-point hedonic scale, where 1 corresponded to “dislike extremely,” 3 to “neither like nor dislike,” and 5 to “like extremely”.

### 3.10. Thermal Analysis

For the investigation of the thermal characteristics of mango jelly, Differential Scanning Calorimetry (TA Instruments DSC Q1000, New Castle, DE, USA) and Thermogravimetric Analyzer (TA Instruments TGA Q500, New Castle, DE, USA) instruments were used. Thermal analysis was performed to evaluate the influence of different processing conditions on the structural and physicochemical stability of the jellies, as processing temperature, sugar concentration, and gelling agents are known to affect the glass transition, crystallization, and degradation behavior of fruit-based gels. These measurements provided complementary information regarding potential differences among M1, M2, and M3 samples, reflecting the effect of high- versus low-temperature regimes on jelly matrix organization and stability. In the DSC experiments, hermetic aluminum pans were utilized, and the sample amount was approximately 3 mg. The samples were first cooled from 20 to −80 °C at a rate of 5 °C/min, followed by heating at the same rate from −80 to 250 °C. Nitrogen flow was set at 50 mL/min. In the case of TGA, the sample mass was approximately 10 mg, and the samples were heated at a rate of 5 °C/min from 25 to 700 °C under a nitrogen flow of 60 mL/min. The TA Universal Analysis 2000 software was used for the analysis of the obtained thermograms.

### 3.11. Statistical Analysis

All experiments were performed in triplicate, and most of the results are expressed as mean ± standard deviation (SD). The obtained data were subjected to analysis of variance (ANOVA) to compare group means. Significant differences between groups were determined using Tukey’s HSD test (*p* < 0.05). Statistical analyses were performed using Statistica v.12 (StatSoft, Inc., Tulsa, OK, USA). Additionally, PCA analysis was performed with Past4Project software (version 4.03), while Pearson’s correlation analysis, presented as a heat map, was generated using Microsoft Excel (version 2016).

## 4. Conclusions

Processing mango fruit under low temperature and vacuum conditions enabled the production of a low-sugar jelly with higher phenolic content and stronger antioxidant activity compared to conventional high-temperature jellies. Importantly, the high fruit content eliminated the need for pectin addition, simplifying formulation while maintaining desirable texture. The low-temperature jellies also demonstrated significant antidiabetic potential by inhibiting both α-amylase and α-glucosidase activity, underlining their promise as functional foods. In general, the superior performance of M2 and M3 in preserving phenolic compounds and antioxidant activity reflects the protective effect of low-temperature vacuum processing, whereas the harsher high-temperature treatment in M1 likely promoted degradation of heat-labile bioactives. Future work should extend this method to other tropical fruits, alone or in combination, and evaluate scalability at pilot and industrial levels. Overall, this processing strategy offers a practical and health-oriented approach to jelly production, supporting the development of clean-label products with enhanced nutritional and functional value.

## Figures and Tables

**Table 1:** Values of TPC (mg GAE/kg jelly ± SD) and VCEAC (mg AAE/100 g jelly ± SD).

Samples	Soluble Solids (%)	TPC (mg GAE/kg Jelly)	DPPH (mg AAE/100 g Jelly)
M1	60.1 ± 0.1 ^a^	70.0 ± 3.7 ^b^	53.0 ± 3.4 ^b^
M2	60.5 ± 0.4 ^a^	82.0 ± 2.0 ^a^	65.0 ± 2.2 ^a^
M3	60.9 ± 0.4 ^a^	80.4 ± 1.9 ^a^	63.3 ± 3.1 ^a^

Each value represents the mean ± SD of three independent replicates. Means with different superscript letters (a, b) are significantly different. Significant differences were determined by one-way ANOVA using the Tukey test, and results were presented as *p*-values (*p* ˂ 0.05). Abbreviations: M1—Jellies prepared under high-temperature regime (boiling before and after sugar/calcium citrate addition); M2—Jellies prepared under low-temperature vacuum regime (55 °C, with calcium citrate, no pectin); M3—Jellies prepared as M2, but with 1% pectin added; TPC—Total phenolic content; GAE—Gallic acid equivalents; DPPH—2,2-diphenyl-1-picrylhydrazyl; AAE—Ascorbic acid equivalents.

**Table 2:** Antioxidant activity measured of jellies by the DC-polarography method (mol reduced Hg(II)/kg jelly ± SD).

Samples	Mol Reduced Hg(II)/kg of Jelly	R^2^
M1	3.74 × 10^−3^ ± 2.6 × 10^−8 b^	0.973
M2	12.40 × 10^−3^ ± 3.0 × 10^−8 a^	0.984
M3	11.84 × 10^−3^ ± 3.0 × 10^−8 a^	0.982

Each value represents the mean ± SD of three independent replicates. Means with different superscript letters (a, b) are significantly different. Significant differences were determined by one-way ANOVA using the Tukey test, and results were presented as *p*-values (*p* ˂ 0.05). Abbreviations: M1—Jellies prepared under high-temperature regime (boiling before and after sugar/calcium citrate addition); M2—Jellies prepared under low-temperature vacuum regime (55 °C, with calcium citrate, no pectin); M3—Jellies prepared as M2, but with 1% pectin added.

**Table 3:** Results of *α-*amylase and *α-*glucosidase enzyme inhibition (% of enzyme inhibition ± SD).

Samples	α-Amylase	α-Glucosidase
M1	n.a.	n.a.
M2	37.50 ± 2.11 ^b^	32.63 ± 7.18 ^c^
M3	1.10 ± 0.15 ^c^	92.88 ± 2.06 ^b^
Acarbose	95.52 ± 4.62 *^,a^	86.26 ± 1.30 *^,a^

Each value represents the mean ± SD of three independent replicates. Means with different superscript letters (a–c) are significantly different. Significant differences were determined by one-way ANOVA using the Tukey test, and results were presented as *p*-values (*p* ˂ 0.05). * the activity of acarbose was determined at 0.5 mg/mL for α-amylase and 1.11 mg/mL for α-glucosidase, while the activity of extracts were tested at 10/11 mg/mL for α-amylase/α-glucosidase, respectively; Abbreviations: M1—Jellies prepared under high-temperature regime (boiling before and after sugar/calcium citrate addition); M2—Jellies prepared under low-temperature vacuum regime (55 °C, with calcium citrate, no pectin); M3—Jellies prepared as M2, but with 1% pectin added; n.a.—non active.

**Table 4:** Sensory evaluation of jellies (mean ± SD).

Samples	Taste	Spreadability	Gelation
M1	4.4 ± 0.4 ^c^	3.9 ± 0.3 ^a^	4.1 ± 0.4 ^b^
M2	4.8 ± 0.4 ^a^	4.0 ± 0.3 ^a^	4.0 ± 0.4 ^b^
M3	4.7 ± 0.5 ^b^	2.7 ± 0.3 ^b^	4.7 ± 0.4 ^a^

Each value represents the mean ± SD obtained from evaluations by 20 volunteers. Means with different superscript letters (a–c) are significantly different. Significant differences were determined by one-way ANOVA using the Tukey test, and results were presented as *p*-values (*p* ˂ 0.05). Sensory attributes were evaluated using a 5-point hedonic scale: 1 = very poor, 2 = poor, 3 = acceptable, 4 = good, 5 = excellent. Abbreviations: M1—Jellies prepared under high-temperature regime (boiling before and after sugar/calcium citrate addition); M2—Jellies prepared under low-temperature vacuum regime (55 °C, with calcium citrate, no pectin); M3—Jellies prepared as M2, but with 1% pectin added.

**Table 5:** Results of thermal analysis of mango jellies.

DSC														
Sample	Cooling				Heating									
	T_on_ (°C)	T_p_ (°C)	T_end_ (°C)	ΔH (J/g)	T_on_ (°C)	T_p_ (°C)	T_end_ (°C)	ΔH (J/g)	T_on_ (°C)	T_p1_ (°C)	T_p2_ (°C)	T_p3_ (°C)	T_end_ (°C)	ΔH (J/g)
M1	−26.1 ± 1.0	−22.5 ± 1.1	−30.6 ± 1.2	59.3 ± 1.6	−10.4 ± 0.1	−2.6 ± 0.1	3.0 ± 0.1	162.3 ± 4.8	111.9 ± 5.6	115.2 ± 4.9	150.1 ± 3.8	171.5 ± 3.8	191.9 ± 2.6	1365 ± 9.8
M2	−29.7 ± 1.0	−30.7 ± 1.2	−37.6 ± 1.8	78.7 ± 2.0	−21.8 ± 1.2	−11.7 ± 1.0	−8.8 ± 0.8	87.6 ± 2.7	102.4 ± 3.8	122.8 ± 4.2	-	202.3 ± 7.2	222.3 ± 6.0	1057 ± 9.9
M3	−29.2 ± 1.0	−29.3 ± 1.3	−32.8 ± 1.0	88.5 ± 1.9	−17.6 ± 1.1	−9 ± 1.0	−4.9 ± 0.2	98.0 ± 2.5	109.8 ± 4.1	131.7 ± 2.5	-	200.6 ± 7.1	221.8 ± 6.9	1018 ± 5.6
TGA														
Sample	WL_1_ (%)	T_s_ (°C)	T_p1_ (°C)	T_p2_ (°C)	T_e_ (°C)	WL_2_ (%)	T_s_ (°C)	T_p_ (°C)	T_e_ (°C)	Res at 700 °C (%)				
M1	64.0 ± 1.8	25.6 ± 1.8	60.8 ± 2.8	-	160.2 ± 4.2	22.1 ± 1.8	160.4 ± 3.5	206.3 ± 4.8	401.3 ± 5.8	10.1 ± 1.8				
M2	46.3 ± 1.5	25.1 ± 0.7	25.3 ± 1.6	110.7 ± 2.8	169.3 ± 4.5	34.7 ± 2.5	169.3 ± 3.8	213.4 ± 3.7	400.7 ± 6.1	13.3 ± 1.2				
M3	47.5 ± 1.6	25.9 ± 0.9	50.2 ± 2.0	115.4 ± 1.9	173.3 ± 5.1	33.4 ± 2.1	173.3 ± 3.9	213.2 ± 2.8	400.4 ± 5.9	12.7 ± 0.8				

with: T_on_—onset temperature, T_p_—peak temperature, T_end_—end-set temperature, ΔH—enthalpy, T_s_—start temperature, T_e_—end temperature, WL—weight loss, Res—residual. Each value represents the mean ± SD of three independent replicates. Abbreviation: M1—Jellies prepared under high-temperature regime (boiling before and after sugar/calcium citrate addition); M2—Jellies prepared under low-temperature vacuum regime (55 °C, with calcium citrate, no pectin); M3—Jellies prepared as M2, but with 1% pectin added.

## Data Availability

Data will be made available on request.

## Supplementary Materials

The following supporting information can be downloaded at https://www.mdpi.com/article/10.3390/ijms262110637/s1.
