# Supplementary material for: A Novel Procedure for Preparing Mango Jellies with Higher Antioxidant Capacity and Reduced Sugar Content
Source: Int J Mol Sci. 2025 Oct 31;26(21):10637. doi: 10.3390/ijms262110637 (PMC12608241; doi:10.3390/ijms262110637)
Supplement: Supplementary file 1 [file ijms-26-10637-s001.zip › ijms-3926043-supplementary.pdf]

International Journal of
Molecular Sciences

MDPI

Supplementary material:

Tables:

Table S1. Reported phenolic compounds in mango (*Mangifera indica* L.) samples from the literature.

Mango sample	Method	Identified phenolic compounds	Reference
Pulp	HPLC–DAD–MS/MS-ESI	Chlorogenic acid, gallic acid, protocatechuic acid, and vanillic acid	Palafox-Carlos et al., 2012
Mango seed karnels, 80% MeOH	HPLC	Cinnamic acid, gallic acid, hesperidin, protocatechuic acid, and tannic acid	Abdel-Aty et al., 2018
Peel powder extracts with liquid nitrogen	HPLC	Ellagic acid, gallic acid, mangiferin, and protocatechuic acid	Jirasuteeruk and Theerakulkait, 2019
Pulp and peel extracts	UPLC–ESI–QTOFMS	Caffeic acid, chlorogenic acid, ferulic acid, gallic, protocatechuic acid, syringic acid, and vanillic acid	Maldonado-Celis et al., 2019
Mango by-products	Review article	Caffeic acid, catechins, chlorogenic acid, coumarin, courmaric acid, ellagic acid, epicatechin, ferulic acid, fisetin, gallic acid, glucogaline, kaempferol, methyl gallate, protocatechuic acid, quercetin, rhamnetin, syringic acid, vanillin	Tirado-Kulieva et al., 2021
70% EtOH leaf extracts identified 15 phenolic compounds	HPLC/DAD and HPLC/ESI-MS Analyses	Gallic acid, galloyl hexose derivative, galloyl hexose isomers 1 and 2, iriflophenone 3-C-(2,6-di-*O*-galloyl) glucoside, iriflophenone 3-C-(2-*O*-galloyl) glucoside, iriflophenone 3-C-glucoside, iriflophenone derivative isomers 1 and 2, maclurin 3-C-glucoside, mangiferin, quercetin 3-*O*-glucoside, quercetin pentoside isomers 1 and 2, and rutin	Sferrazzo et al., 2022
leaves extracts	UPLC-HRMS	Di-*O*-methylquercetin, gallic acid, kaempferol, mangiferin, naringenin-*O*-hexoside, *O*-methylnaringenin pentoside, protocatechuic acid, trihydroxybenzoyl mangiferin	Selim et al., 2024
leaves EtOH extract	LC–MS/MS	Acetohydroxamic acid, butein, caffeic acid, catechin hydrate, fumaric acid, gallic acid, myricetin, phloridzin dihydrate, quercetin, resveratrol, and vanillic acid	Işık et al., 2025

Table S2. Pearson's coefficient of the correlation matrix of the examined parameters in mango jellies presented as correlation between quantified total phenolic content (TPC), and applied antioxidant assay (DPPH) and antidiabetic (alpha-amylase and alpha-glucosidase) assays. Green squares represent a highly significant correlation of inspected parameters, while red squares present low interactions, assessed according to the corresponding Pearson's coefficient.

	TPC	DPPH	α-amylase	α-glucosidase
TPC	1.00			
DPPH	0.99	1.00		
α-amylase	0.38	0.31	1.00	
α-glucosidase	-0.98	-0.94	-0.42	1.00

References

Abdel-Aty, A.M.; Salama, W.H.; Hamed, M.B.; Fahmy, A.S.; Mohamed, S.A. Phenolic-antioxidant capacity of mango seed kernels: therapeutic effect against viper venoms. *Rev. Bras. Farmacogn*. **2018**, *28*, 594–601. https://doi.org/10.1016/j.bjp.2018.06.008

Işık, M.; Dikici, E.; Altın, S.; Alp, C.; Kırboğa, K.K.; Köksal, E.; Beydemir, Ş. Phenolic content, antioxidant capacity, and therapeutic potential of mango (*Mangifera indica* L.) leaves. *Food Sci. Nutr*. **2025**, *13*(5), e70263. https://doi.org/1002/fsn3.70263

Jirasuteeruk, C.; Theerakulkait, C. Ultrasound-assisted extraction of phenolic compounds from mango (*Mangifera indica* cv. Chok Anan) peel and its inhibitory effect on enzymatic browning of potato puree. *Food Technol. Biotechnol.* **2019**, *57*(3), 350. https://doi.org/10.17113/ftb.57.03.19.5728

Maldonado-Celis, M.E.; Yahia, E.M.; Bedoya, R.; Landázuri, P.; Loango, N.; Aguillón, J.; Restrepo, B.; Guerrero Ospina, J.C. Chemical composition of mango (*Mangifera indica* L.) fruit: nutritional and phytochemical compounds. *Front. Plant Sci*. **2019**, *7*(10), 1073. https://doi.org/10.3389/fpls.2019.01073

Palafox-Carlos, H.; Yahia, E.M.; González-Aguilar, G.A. Identification and quantification of major phenolic compounds from mango (*Mangifera indica*, cv. Ataulfo) fruit by HPLC–DAD–MS/MS-ESI and their individual contribution to the antioxidant activity during ripening. *Food Chem.* **2012**, *135*(1), 105–111. https://doi.org/10.1016/j.foodchem.2012.04.103

Selim, D.A.; Shams Eldin, S.M.; González, F.P.; Ghareeb, D.A.; Shawky, E. Metabolomics and chemometrics-driven valorisation of mango leaves: Unveiling putative α-amylase and α-glycosidase inhibitory metabolites. *Food Biosci.* **2024**, *61*, 104652, https://doi.org/10.1016/j.fbio.2024.104652

Sferrazzo, G.; Palmeri, R.; Restuccia, C.; Parafati, L.; Siracusa, L.; Spampinato, M.; Carota, G.; Distefano, A.; Di Rosa, M.; Tomasello, B.; Costantino, A.; Gulisano, M.; Li Volti, G.; Barbagallo, I. *Mangifera indica* L. leaves as a potential food source of phenolic compounds with biological activity. *Antioxidants* **2022**, *11*(7), 1313. https://doi.org/10.3390/antiox11071313

Tirado-Kulieva, V.; Atoche-Dioses, S.; Hernández-Martínez, E. Phenolic compounds of mango (*Mangifera indica*) by-products: Antioxidant and antimicrobial potential, use in disease prevention and food industry, methods of extraction and microencapsulation. *Sci. Agropecu*. **2021**, *12*(2), 283–293. https://doi.org/10.17268/sci.agropecu.2021.031

Disclaimer/Publisher's Note: The statements, opinions and data contained in all publications are solely those of the individual author(s) and contributor(s) and not of MDPI and/or the editor(s). MDPI and/or the editor(s) disclaim responsibility for any injury to people or property resulting from any ideas, methods, instructions or products referred to in the content.
